# Ulcerative Necrobiosis Lipoidica: Is There a Place for Anti-TNF*α* Treatment?

**DOI:** 10.1155/2012/854738

**Published:** 2012-05-23

**Authors:** Rita Guedes, Inês Leite, Armando Baptista, Natividade Rocha

**Affiliations:** Dermatology Department, Centro Hospitalar de Vila Nova de Gaia Espinho, Rua Conceição Fernandes, 4434-502 Vila Nova de Giaa, Portugal

## Abstract

Necrobiosis lipoidica is a rare granulomatous and inflammatory disease. Its management is particularly difficult when ulceration is present. The authors describe the clinical case of a 65-year-old female patient with necrobiosis lipoidica, who had been submitted in the past to several topical and systemic treatments with little or no improvement. She started treatment with subcutaneous etanercept and showed significant improvement without adverse events until today. The aim of this article is to report a valid and efficient alternative treatment to recalcitrant cases.

## 1. Introduction

Necrobiosis lipoidica (NL) is a rare, idiopathic, chronic granulomatous inflammatory disorder. Ulceration occurs in up to 35% of cases [1], leading to greater risk for secondary infection and, in rare cases, to squamous cell carcinoma. Until today there is no standardized effective treatment.

**Figure 1:**
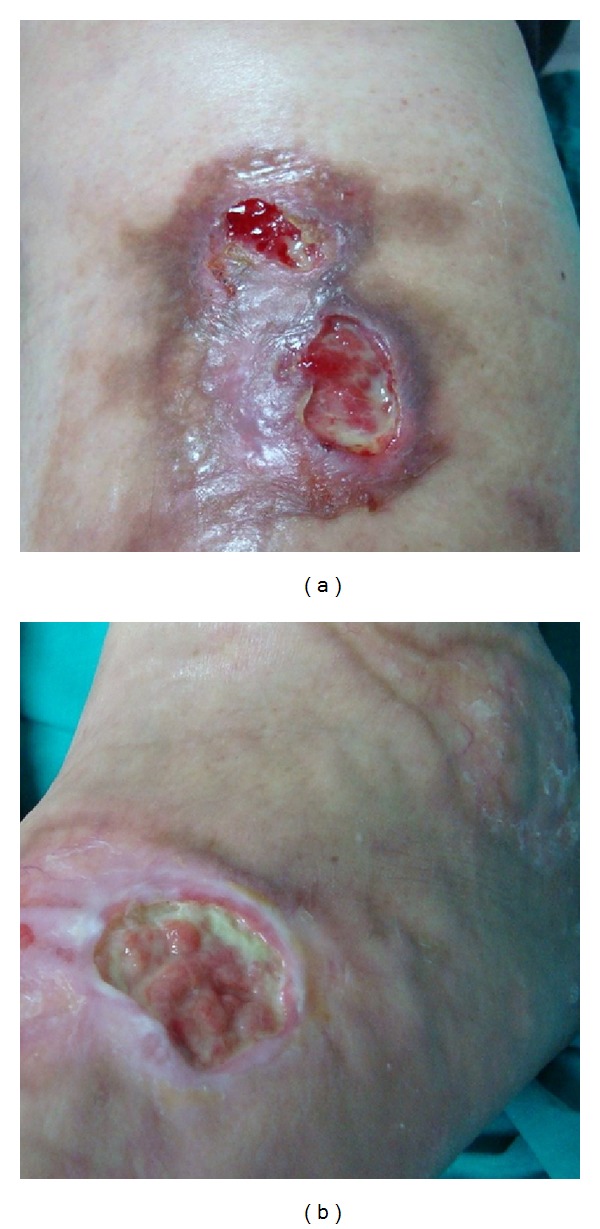
Ulcerative lesions on the patients' shins (a) and feet (b).

We describe the clinical case of a 65-year-old female, type 2 diabetic patient, with a body mass index of 31,2 and a clinical and histological diagnosis of necrobiosis lipoidica for 6 years (Figures 1(a) and 1(b)).

**Figure 2:**
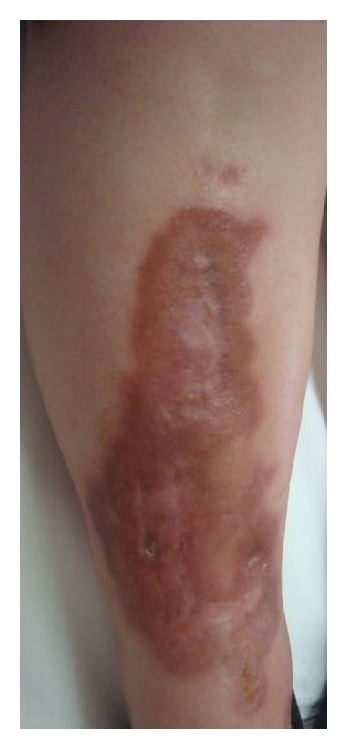
Healed lesion after 12 weeks of therapy.

She had been previously submitted to several topical and systemic treatments, namely, potent topical corticoids, topical 0.1% tacrolimus, and systemic cyclosporine (3 mg/kg/day). Topical corticoids and tacrolimus were ineffective. After 4 months of therapy with cyclosporine, she achieved complete resolution of her lesions. However, due to renal toxicity, the drug had to be stopped, with the recurrence of the lesions. Four months later, we started etanercept (50 mg/week) subcutaneously and suspended all the topic treatments done until then. At 8 weeks of therapy, she had all her ulcerative lesions healed and the borders of the nonulcerated lesions were less infiltrated (Figure 2). To date, she completed 6 months of 50 mg weekly etanercept with no adverse events.

## 2. Comment

Treatments for NL have had limited success, due either to the unclear pathogenesis of the disease [2] or to significant side effects associated with effective treatments, namely, worsening of diabetes with oral corticoids, renal toxicity with cyclosporine, medullar toxicity with mycophenolate mofetil, and peripheral neuropathy with thalidomide. Moreover, the relatively rarity of the disease makes randomized studies difficult to perform. Although many drugs have been used in this pathology, none has demonstrated to be truly effective. Ulcerative lesions in diabetic patients, in particular, can be a challenge, due to the pain associated with the topical application of some agents or dressings and also due to the scarce vascularity of the lesions, making reepithelization difficult.

TNF-*α* is an inflammatory cytokine with a role in cellular differentiation, mitogenesis, cytotoxicity, inflammation, immunomodulation, and wound scarring. It seems to be particularly important in the development of granulomatous inflammatory diseases, like NL. The inhibition of this cytokine can be achieved with the use of a new drug class, which at this moment is approved for rheumatoid arthritis, psoriatic arthritis, ankylosing spondylitis, juvenile idiopathic arthritis, and psoriasis. Besides their official indications, these drugs have been used with promising results in other diseases [3].

Necrobiosis lipoidica nonresponsive to other therapeutic agents, as a granulomatous disease, has been treated with this drug class since 2003 [4]. Infliximab was the first anti TNF-*α* drug to be prescribed in NL [4]. However, due to intravenous administration and frequent development of neutralizing antibodies, one may prefer other options in the same drug class. Nonulcerating NL treated with etanercept has already been reported [2, 3, 5, 6] both with subcutaneous and with intralesional injection; however, reports about the treatment of ulcerative lesions are scarce. Suárez-Amor et al. reported the successful treatment with etanercept of an ulcerative NL patient nonresponsive to other first-line treatments [1], demonstrating that this drug can be an alternative drug in resistant cases.

Our case reports the treatment of ulcerative NL with etanercept, showing a superior effect compared to cyclosporine. Healing of the ulcerative lesions occurred during a shorter period of therapy and with a favourable safety profile, showing that it can be a valid option in cases in which cyclosporine is contraindicated. The authors are aware of possible side effects of the drug, namely, the potential risk of increased malignancies. Regarding necrobiosis lipoidica etanercept could potentiate the transformation to squamous cell carcinoma. However, due to the lack of response of former treatments, this drug should be offered to patients, after explaining them these possible side effects.

Therapeutic results presented in this clinical report raise new questions about TNF-*α*'s role in the disease and suggest a therapeutic alternative for resistant cases to classic treatments. New reports describing the management of more patients will help to optimize therapeutic strategies.

## References

[1] Suárez-Amor O, Pérez-Bustillo A, Ruiz-González I, Rodríguez-Prieto MA (2010). Necrobiosis lipoidica therapy with biologicals: an ulcerated case responding to etanercept and a review of the literature. *Dermatology*.

[2] Zhang KS, Quan LT, Hsu S (2009). Treatment of necrobiosis lipoidica with etanercept and adalimumab. *Dermatology Online Journal*.

[3] Alexis AF, Strober BE (2005). Off-label dermatologic uses of anti-TNF-a therapies. *Journal of Cutaneous Medicine and Surgery*.

[4] Kolde G, Muche JM, Schulze P, Fischer P, Lichey J (2003). Infliximab: a promising new treatment option for ulcerated necrobiosis lipoidica. *Dermatology*.

[5] Zeichner JA, Stern DWK, Lebwohl M (2006). Treatment of necrobiosis lipoidica with the tumor necrosis factor antagonist etanercept. *Journal of the American Academy of Dermatology*.

[6] Cummins DL, Hiatt KM, Mimouni D, Vander Kolk CA, Cohen BA, Nousari CH (2004). Generalized necrobiosis lipoidica treated with a combination of split-thickness autografting and immunomodulatory therapy. *International Journal of Dermatology*.

